# Supplementary material for: Real-world effectiveness of a community-based multicomponent maternal smoking cessation program in preventing low birthweight deliveries: Findings from the CTTP cohort
Source: Tob Induc Dis. 2025 Oct 24;23:10.18332/tid/210321. doi: 10.18332/tid/210321 (PMC12551384; doi:10.18332/tid/210321)
Supplement: Supplementary file 1 [file TID-23-163-s1.pdf]

Supplementary file Table 1

Variable	Missing (%)
Enrollment age	0.21
Trimester	0.64
Cigarettes smoked at Enrollment	0.43
Race/Ethnicity	0.07
Household Smoking	0.00
Prolonged Abstinence	0.14
Point Prevalence Abstinence at 2-4 months	0.00
Point Prevalence Abstinence at 6 months	0.00
Pre-term Birth	45.51
Low Birthweight	49.50
NICU admission	53.71

© 2025 Ahmed S.D. et al.
